# Toward Child-Centred Artificial Intelligence in Pediatric Emergency Medicine: A Perspective on Clinical Decision Support, Stakeholder Engagement and Education

**DOI:** 10.3390/pediatric18040091

**Published:** 2026-07-08

**Authors:** Lorenzo Gasparini, Nicola Gobbi, Daniele Zama, Marcello Lanari

**Affiliations:** 1 Specialty School of Paediatrics, Alma Mater Studiorum, University of Bologna, 40138 Bologna, Italy; 2 Department of Medical and Surgical Sciences, Alma Mater Studiorum, University of Bologna, 40138 Bologna, Italy; 3 Pediatric Emergency Unit, IRCCS Azienda Ospedaliero-Universitaria di Bologna, 40138 Bologna, Italy

**Keywords:** pediatric, emergency department, artificial intelligence, machine learning, stakeholder, clinical decision support, education

## Abstract

Artificial intelligence (AI) is increasingly recognized as a transformative technology in healthcare, with growing evidence supporting its applicability across time-critical clinical environments. This perspective aims to evaluate the integration of AI and machine learning (ML) into pediatric emergency departments (PEDs) across three core domains: clinical decision support, stakeholder engagement, and medical education. Within clinical decision support, ML architectures have demonstrated high predictive performance across several high-acuity clinical scenarios, including triage stratification, pediatric traumatic brain injury risk classification, early sepsis detection and clinical deterioration prediction, and dermatological assessment. Model interpretability and real-world implementability remain critical prerequisites for clinical adoption, with explainability methods representing fundamental instruments to enhance transparency and stakeholder trust. Regarding stakeholder engagement, the triadic dynamic among clinicians, caregivers, and patients defines a unique communication challenge in PEDs, with large language models (LLMs) showing preliminary utility; however, stakeholder-inclusive model validation and robust data privacy protections for minors remain key challenges, particularly regarding legal ambiguities of LLM deployment in clinical pipelines. In medical education, AI-driven simulation platforms and LLM-generated adaptive curricula represent promising tools for competency-based training across pediatric emergency scenarios. Future directions emphasize the imperative of prospective multicenter validation in pediatric-specific cohorts, rigorous data quality standards addressing conformance, completeness, and plausibility, and the development of pediatric-tailored governance frameworks. Real-world implementation will require the systematic involvement of all stakeholders—including children, caregivers, clinicians, developers, and institutions—as co-designers of equitable, transparent, and safe AI systems for this uniquely vulnerable population.

## 1. Introduction

Pediatric emergency departments (PEDs) are time-sensitive settings where the unique needs of pediatric patients necessitate tailored clinical pathways. Within an overcrowded emergency field, the ability to detect potential rapid clinical deterioration is crucial, from triage through to reassessment by clinicians. In addition to managing urgent and life-threatening conditions, PEDs also face a substantial volume of low-acuity cases with highly heterogeneous presentations, many of which could benefit from innovative tools to improve the efficiency and effectiveness of diagnostic and therapeutic management. In the last decade, digital solutions such as Electronic Health Records (EHRs) have been integrated into clinical management. These have proven to be essential tools for clinical decision support while simultaneously acting as a catalyst for leveraging massive datasets, driving the spread of Artificial Intelligence (AI) in healthcare [1,2]. The aim of this study is to evaluate the integration of AI systems into the clinical practice of PEDs, focusing on potential applications, stakeholder involvement, education and training, and current limitations.

## 2. Definitions

AI is a technology that empowers computers and machines to simulate human behavior and cognitive functions [1].

### 2.1. Machine Learning (ML)

ML is a process of using large datasets to train a model and enable it to “learn” patterns and relationships within the data. This learning is not intuitive but it is fundamentally a mathematical optimization process. Model training is the core phase of the ML pipeline, in which the model’s parameters—the numerical values, known as weights and biases, that govern its outputs—are iteratively adjusted to minimize a loss function, a measure of how far the model’s predictions deviate from the expected results. When this loss falls below a predefined threshold, the model is considered trained [1,3]. Within this framework, several learning paradigms are commonly used [3]:

1. Supervised machine learning (SL) is a technique that uses labeled training datasets to define the relationships between input and output data. Three phases can be distinguished: training, evaluation, and cross-validation. A wide range of machine learning algorithms excel in supervised learning, including linear regression, logistic regression, Naïve Bayes, polynomial regression, Support Vector Machines (SVM), K-nearest neighbors (KNN), and Random Forests (RF) [1].
2. Unsupervised learning is a technique that analyzes and clusters unlabeled data, discovering hidden patterns or groupings without an external ground truth [1].
3. Reinforcement learning (RL) differs from these paradigms by optimizing sequential decision-making through trial and error, where models learn policies that maximize a reward function rather than minimizing prediction error [4].

### 2.2. Deep Learning (DL)

DL is a subset of ML based on multilayered neural networks inspired by the structure of the human brain. The “multilayered” designation refers to structure of at least three layers—an input layer, one or more hidden layers, and an output layer—where even a single hidden layer constitutes basic depth; modern architectural pipelines, however, routinely employ significantly more. Each node, or “neuron,” in the stacked layers performs a mathematical operation; this intricate series of nested operations maps an input to an output. DL models are particularly powerful at learning complex and highly non-linear relationships in data, a feature especially evident when handling large volumes of inputs. Among the different types of deep learning architectures are Convolutional Neural Networks (CNNs) or Recurrent Neural Networks (RNNs) [1,5].

#### Large Language Models (LLMs)

LLMs are a category of DL and the principal driver of generative AI. Trained on billions of data points, LLMs are built on neural network architecture and operate by predicting the next word in a sequence, learning patterns to generate coherent and contextually relevant text. They are readily accessible through interfaces such as ChatGPT, Claude, Gemini, Llama, and Copilot, among others. Similarly, text-to-image systems such as DALL-E, Midjourney, and Stable Diffusion use specific generative adversarial networks to produce images from text [2].

### 2.3. Explainable AI (XAI)

In PED, ML models applied to EHR and monitoring data have demonstrated promising performance across a broad spectrum of clinical decision support applications, these models may improve upon traditional scoring systems by capturing complex nonlinear relationships. However, limited interpretability remains a major barrier to clinical adoption. One of the most promising subsets in the AI landscape is, undoubtedly, Explainable Artificial Intelligence (XAI) that may help improve the transparency, safety, equity, and interpretability of DL models, enabling physicians to comprehend, validate, and provide continuous feedback to AI systems [6]. The most common XAI methods include Shapley Additive ExPlanations (SHAP), Local Interpretable Model-agnostic Explanation (LIME) and RETAIN. Providing specific explanations and assigning quantifiable values to each variable influencing a clinical presentation may improve physician confidence in adopting using ML models [6].

## 3. Applications

### 3.1. Diagnostic, Clinical and Therapeutic Decision Support

#### 3.1.1. Enhancing Triage

Triage assessment in emergency care systems is increasingly challenging due to rising patient volumes and overcrowding. In the PEDs, triage represents a critical initial step aimed at stratifying patients according to clinical severity, resource requirements, urgency of treatment, and risk of deterioration. The primary goal is the early identification of potentially critically ill children to optimize resource allocation and ensure high-quality care delivery. Importantly, triage is not intended to predict patient outcomes. Minimizing both undertriage and overtriage is essential to maintain patient safety and system efficiency. Overcrowding in PED is associated with prolonged waiting times, reduced quality of care, increased risk of medical errors, extended length of stay and potential threats to patient safety. The integration of EHRs in PED represents a transformative shift and paves the way to ML algorithms and AI application for triaging. While traditional methods often rely on linear models and limited variables, ML algorithms, such as Random Forest, XGBoost, and Deep Neural Networks, can process high-order nonlinear interactions among a multitude of predictors extracted from [7]. ML-based triage systems represent a promising strategy to improve patient stratification, reduce PED overcrowding and enhance clinical outcomes. Literature highlights variable performance and current limitations of AI-based triage systems in PED. In 2020, Goto et al. [8] applied four machine learning models—Lasso Regression, RF, Gradient-Boosted decision tree, and deep neural network (DNN)—to a nationally representative sample of 52,037 PED visits (2007–2015), demonstrating superior discriminative ability compared to conventional triage for predicting critical care needs and hospitalization. In particular, all used ML models showed a better discrimination for critical care needs, which occurred in 0.3% of the patients, (e.g., deep neural network sensitivity 0.78 vs. 0.54 for conventional triage), reducing the risk of undertriage of children who may rapidly deteriorate. This gain in sensitivity reflects low positive predictive value (PPV 0.01 [95% CI, 0.01–0.02]) and high negative predictive value (NPV 0.99 [95% CI, 0.99–0.99]), whereas conventional triage achieved the highest positive likelihood ratio (PLR 5.72 [95% CI, 4.29–7.64]), but failed to detect most of the critical care needs. For hospitalization prediction, which occurred in 4.5% of the patients, all the ML models achieved greater specificity (e.g., lasso regression specificity 0.75 vs. 0.55), and higher PLR, leading to fewer overtriage. Overall discrimination improved significantly, with area-under-the-curve (AUC) reaching 0.85 for critical care and 0.80 for hospitalization prediction (vs. 0.78 and 0.73 for conventional triage, respectively), supporting ML as a promising decision-support tool in PED. Nevertheless, these promising results must be contextualized within the study’s limitations, including its retrospective design, its high-income national setting, and additional constraints transparently reported by the authors, which may affect external validity [8]. Sánchez-Salmerón et al. conducted a comprehensive study of ML approaches used in emergency triage: deep neural network achieved the best prediction results, followed by XGBoost and random forest, while traditional methods like logistic regression lag behind [9]. The integration of natural language processing (NLP) techniques, including transformer-based models, further enhances the analysis of unstructured clinical data. Despite these advancements, key challenges remain, particularly regarding model interpretability and clinical applicability. Complex ML models, although highly accurate, often lack transparency, which limits their adoption in real-world settings. The implementation of XAI frameworks, such as SHAP and LIME, is therefore essential to improve trust and usability. However, further prospective, multicenter cross-validation, especially in pediatric populations, is required before widespread clinical implementation [10].

#### 3.1.2. Trauma Care: Pediatric Traumatic Brain Injury (pTBI)

Pediatric traumatic brain injury (pTBI) affects thousands of children every year and is cause of disability and death worldwide. The estimated global incidence of pTBI is 226.4/100.000 children, equivalent to at least 5 million new pTBI cases annually among individuals aged 0–18 years, with a mortality rate from 0.3% in high-income countries to a 13.5% in low-income countries [11]. Despite the large numbers of children who experience TBI, only a small percentage requires hospitalization or prolonged surveillance. The introduction in daily practice of clinical decision rules such as PECARN, CATCH, and CHALICE with high sensitivity to differentiate between patients with mild pTBI that need for an imaging has reduced the number of unnecessary CT scans in pediatric patients [12]. Despite this, the specificity and positive predictive values of these methods are low, with most children undergoing CT scans having no findings of intracranial injury. Thus, the creation of AI tools for identifying patients at risk for clinically relevant TBI (CRTBI) could provide a mechanism for early safe discharge and potentially reduce unnecessary healthcare expenditures and x-ray exposition in a vulnerable phase of life [13]. A recent systematic review on the use of ML in pTBI highlighted that classical supervised algorithms—including artificial neural networks (ANN), RF, SVM, and KNN—are the most frequently applied models. Reported performances were high, with maximum AUC values of 99.1% for outcome prediction and 98.0% for identifying the need for CT imaging; despite these promising reported performances, it is important to highlight that several methodological concerns—including reliance on single retrospective datasets, relatively small sample sizes within clinically relevant pTBI subgroups, variability in radiological interpretation, and lack of external validation—may compromise model robustness and inflate performance metrics through overfitting [14,15]. SVM models, like the one by Tunthanathip et al., and ANN model by Hale et al. demonstrated excellent predictive accuracy (94.0% and AUC = 0.991, respectively). Despite these promising results, the clinical adoption of ML in pTBI remains limited due to heterogeneous data, insufficient external validation, and concerns regarding generalizability [15,16]. Nevertheless, the superior performance of some ML models compared to traditional clinical decision rules such as PECARN suggests their potential to enhance early identification and management of clinically relevant pediatric TBI, optimizing clinical care, radiation exposure, and resource allocation [17].

#### 3.1.3. Dermatology in PED

Skin disorders represent one of most common causes of access to the PED with a wide spectrum of dermatological findings that range from allergic/inflammatory reactions to skin infections, congenital disorders, or chronic skin disorders. Although this necessity exists, pediatric dermatology is one of the most underserved sub-specialties in childhood medicine. Skin disorders in children require a tailored approach, and the application of AI in pediatric dermatology needs more studies investigating pediatric cohorts. Deep learning methods, such as CNNs, have proven effective in identifying certain pediatric skin conditions, such as infantile hemangioma; however, to date, no DL-applications have been validated for pediatric dermatology in the pediatric emergency setting. AI-driven applications and platforms may facilitate remote consultations between pediatric dermatologists and emergency physician, acting as a point-of-care solution in underserved areas and enabling preliminary clinical assessments [18]. Recently, Gençeli et al. [19] conducted a comparative diagnostic accuracy study evaluating three LLMs models—ChatGPT (GPT-5), Gemini 3 Pro, and Microsoft Copilot—against a stratified cohort of 263 pediatric clinicians (107 residents, 156 specialists) across 61 cases of exanthematous diseases spanning 23 distinct conditions, using a standardized format consisting of a clinical photograph paired with structured clinical data. ChatGPT and Gemini—achieved high diagnostic accuracy of 86.9% and 82.0%, respectively, both exceeding the upper bound of the specialist population diagnostic performance, while all three models surpassed the resident median performance Disease-level subanalyses revealed a consistent pattern of failure in context-dependent diagnoses: all three models achieved 0% accuracy for insect bites, and scabies accuracy ranged from 40% to 80%, suggesting that LLMs performance degrades when correct identification requires integration of exposure history, lesion distribution, or epidemiologic clues [19]. Although current-generation LLMs demonstrate promising diagnostic performance for morphologically distinct pediatric exanthems, real-world implementation within pediatric emergency workflows remains an ambitious but achievable goal, contingent upon addressing key gaps that current evidence has clearly delineated [19]. Future research should prioritize cross-validation encompassing the full spectrum of pediatric populations—including Fitzpatrick skin phototypes I, V, and VI—and invest in the development of standardized, safety and reproducible prompting protocols; fulfilling these requirements would support the deployment of LLMs as validated clinical decision support tools in the pediatric emergency setting, expanding diagnostic equity across underrepresented and underserved populations.

#### 3.1.4. Pediatric Sepsis and Intensive Care Access

Sepsis in children is associated with substantial morbidity and mortality, with effects that may be severe, heterogeneous, and long-lasting. Outcomes vary considerably according to geographic setting, access to health care, and resource availability, contributing to poor short and long-term prognosis [20]. Already in primary care settings, general free-to-use LLMs’ performance has been tested in providing appropriate, detailed and applicable antibiotic prescriptions, analyzing antibiograms and predicting neonatal sepsis [21], showing overall acceptable accuracy, completeness and clinical translatability, in particular for standardized diagnoses and well-defined guidelines [22]. Published prediction models and scoring systems incorporate combinations of clinical, laboratory, and physiologic variables and, in some cases, novel biomarkers. Short-term pediatric models, including the Pediatric Sepsis Biomarker Risk Model (PERSEVERE), which uses five biomarkers to estimate 28-day mortality and persistent organ dysfunction in septic shock [23], and the Pediatric Logistic Organ Dysfunction (PELOD-2 score), primarily focus on early deterioration, mortality, organ dysfunction, and length of stay. However, their utility for long-term outcome prediction appears limited, as suggested by the lack of consistent associations between commonly used predictors and subsequent impairment in health-related quality of life (HRQoL) or disease trajectory [24]. ML approaches offer potential advantages in pediatric sepsis and critical care. These include earlier recognition of clinical deterioration, enabling timely interventions, and the ability to identify clinically relevant phenotypes to support more individualized management. ML models can leverage routinely collected EHR and bedside monitoring systems to analyze large volumes of clinical information and inform clinical decision-making. Current applications include early detection of critical conditions (e.g., sepsis and bloodstream infections), risk stratification, prediction of outcomes (e.g., mortality and length of stay), and clinical decision support [25]. In 2024, the Pediatric Sepsis Definition Task Force has developed and validated the Phoenix Sepsis Score—an integer-based tool assessing respiratory, cardiovascular, coagulation, and neurologic dysfunction—using EHR-based dataset from over 3.6 million pediatric encounters across 10 international sites both in high- and low-resource settings. The Phoenix Sepsis Score demonstrated better diagnostic performance for pediatric sepsis and septic shock compared to previous criteria. For the score’s development, an interpretable ML approach specifically named stacked regression was used. Stacked regression, a robust model-averaging approach, simultaneously combined the best-performing organ dysfunction sub-scores—drawn from different previous existing scores—as input variables, estimating each subcomponent’s relative contribution to mortality prediction according to its predictive power, while maintaining a high degree of clinical interpretability [26]. In addition, ML may contribute to advances in precision medicine by identifying subgroups more likely to benefit from targeted therapies. For example, Sturtz et al. developed and externally validated the pediatric Critical Event Risk Evaluation and Scoring Tool (pCREST), based on an xGBoost model, which demonstrated improved performance compared with DL approaches in predicting the likelihood of a critical event within 12 h of any recorded vital sign or laboratory measurement during hospitalization [27]. In a retrospective study evaluating ML algorithms to predict PICU length of stay, Ganatra et al. reported that models including gradient boosting, CatBoost, and RNNs achieved moderate predictive performance, with accuracy slightly exceeding 70%, outperforming clinician-based estimates [28]. In the context of pediatric sepsis and critical care, the routine collection of large volumes of clinical data makes ML integration into clinical practice an attainable goal. However, ensuring model generalizability will require the systematic inclusion of data from both high- and low-resource settings. Federated learning frameworks may offer a viable solution to this challenge, allowing model development across distributed datasets while maintaining data privacy and data integrity.

### 3.2. Stakeholders Involvement

The integration of LLMs into PED demands a nuanced understanding of the unique stakeholder landscape that characterizes pediatric care. Unlike adult settings, PED involve a triadic dynamic between clinicians, patients, and proxy stakeholders—predominantly parents or minor’s legal representatives—whose health literacy, language background, and emotional state directly shape the quality of clinical encounters. A recent scoping review of LLMs applied to clinical text in pediatrics highlighted that family- or caregiver-facing applications remain substantially underrepresented in the literature, despite the central mediating role that these figures play in pediatric care delivery. Specifically, Huang et al. found that none of the 40 included studies engaged parents, caregivers, or patients as stakeholders in study design, model development, or evaluation, and that only a minority of studies addressed administrative or communication-oriented LLM applications—such as summarization or patient-facing messaging—compared to the dominant focus on diagnostic decision support [29]. This is particularly relevant in PEDs, where caregivers function as the primary interlocutors between the clinical team and the child, communicating symptoms, providing medical history, and subsequently implementing discharge instructions at home; the absence of caregiver perspectives from the design of AI communication tools therefore represents not merely a methodological gap but a potential patient safety concern. Linguistic barriers represent a particularly critical challenge in this context and a significant vector for reduced quality of care in emergency department. Ray et al. [30] tried to explore the potential of LLM-generated translations of patient discharge instructions into Spanish and whether those translations could be comparable in quality to professional human translators. The study showed clinical promise but also meaningful variability in accuracy, illustrating the risk that automated communication tools may introduce when caregivers of non-English-speaking families are the primary recipients of safety-critical information [30]. The distinct communicative demands of PEDs, where discharge instructions must simultaneously address the caregiver’s comprehension needs and—where developmentally appropriate—the child’s own understanding, require a dual-audience communication strategy that current LLM evaluation frameworks have yet to systematically assess. Consequences of miscommunication in this context are not insignificant: inaccurate or misunderstood discharge instructions may result in failure to recognize clinical deterioration, incorrect medication administration, or delayed return to care, all of which carry potentially serious clinical consequences for a population that is inherently vulnerable and dependent on adult proxies for health decision-making. However, the deployment of such patient-facing tools raises acute data privacy concerns that cannot be overlooked. In the US, the Health Insurance Portability and Accountability Act (HIPAA) supports specific de-identification rules for clinical data, with expert consensus as an alternative approach better matched to AI/ML linkage risk (e.g., high-dimensional EHRs, imaging metadata); in Europe, the GDPR establishes a high anonymization threshold, while residual risks from pseudonymous data and indirect identifiers persist, requiring explicit consent from the patient, parent, or legal guardian. Compliance is further complicated by the fact that many commercially available LLM platforms were not designed with healthcare data protection requirements in mind, and the boundaries of business associate agreements in this context remain legally ambiguous [31]. The scale of this risk is further underscored by Huang et al. [29], who found that 85% of included pediatric studies did not indicate whether HIPAA-compliant models were used, and that the frequent use of publicly hosted LLM platforms—which do not operate in HIPAA-compliant environments—further elevates the risk to sensitive patient data. In the context of PED, where parental consent, minor patient confidentiality, and the urgency of clinical encounters create a uniquely complex privacy landscape, these regulatory gaps are especially concerning. Furthermore, challenges related to demographic underrepresentation within training datasets risk compounding existing health disparities: equity considerations are particularly important in children given their increased vulnerability, the cumulative effects of early-life disparities, and the potential for preventive intervention [29]. Hogg et al. [29,32] proposed a systematic review of stakeholder perspectives on clinical AI, finding that trust, transparency, and perceived clinical benefit are fundamental prerequisites for adoption across all stakeholder groups; they considered five different stakeholder groups: developers, health care professionals, health care managers and leaders, patients—including carers, families and the public—and regulators and policy makers, all of which are necessary for AI implementation so that their exclusion from design and deployment processes is a recurrent barrier to sustainable integration. In this study, they highlighted that in clinical AI literature, there is a strong representation of healthcare professionals’ (HCPs’) perspectives; instead, non-HCP stakeholders account for only 30% of available evidence, with carer-specific perspectives representing a negligible 0.35% of the total. This clinician-centric bias may lead to a comprehensive understanding of the multistakeholder dynamics that drive successful AI implementation. On a more encouraging note, implementation factors appear largely consistent across both rule-based and non-rule-based AI tools, suggesting that the broader clinical AI evidence base can meaningfully inform the deployment of more modern, non-rule-based systems. Overall, the authors call for a more inclusive research agenda that prioritizes underrepresented stakeholder voices and addresses the unique implementation challenges posed by contemporary AI technologies [29,32]. Overall, these findings underscore the urgent need for a participatory, equity-oriented, and privacy-conscious approach to ML integration in PED, one that meaningfully involves parents and caregivers throughout model development, addresses linguistic and health literacy barriers through validated multilingual outputs such as those evaluated by Ray et al. [30], and adheres strictly to regulatory frameworks governing the protection of children’s health data as outlined by Marks and Haupt [31].

### 3.3. Education and Training

Simulation-based team training has been firmly established as an essential pillar of pediatric emergency education, supporting both technical skill acquisition and non-technical competencies such as team communication and clinical decision-making in high-acuity scenarios—making it a natural foundation upon which emerging ML and artificial intelligence tools can build [33]. The integration of ML into pediatric medical education represents a significant shift in how physicians, trainees, and institutional stakeholders approach training and competency development, with LLMs showing particular promise in benefiting medical students, residents, fellows, practicing pediatricians, and advanced practice providers; notably, when performance evaluations are uploaded, LLMs can generate individualized learning plans targeting gaps in knowledge, communication, or clinical reasoning, and can build evaluation tools and competency-based assessments useful for program directors and course directors [34]. The SaNuRN program, reviewed by Grosjean et al., demonstrates the educational potential of AI, specifically NLPs, in simulated training through a virtual clinical simulator; the program is enabled to act as either the patient or the professional, providing questions and responses accordingly, leading providers in a low-stakes environment and allows them to view both perspectives [35]. In the specific domain of pediatric sepsis—a condition where outcomes depend on timely recognition and rigorous management, and where traditional teacher-centered approaches have repeatedly demonstrated knowledge gaps among trainees—AI-driven tools hold particular promise for enhancing the full spectrum of educational methods, from augmenting gamified platforms with dynamic scenario adjustment to managing simulation flow in real time, providing intelligent debriefing insights, and enabling natural language interactions in virtual reality environments, with direct implications for multidisciplinary teams and quality improvement stakeholders at the institutional level [36]. For example, a human-centered AI enabled training frameworks for emergency response, LEARNER platform, insisting of three core components—actionable measures, adaptable elements, and a guiding strategy— utilizes physiological and behavioral (PNB) markers, collected via wearable sensors, self-reports, and interaction behaviors, to tailor the learning curriculum to each individual’s unique educational needs [37]. The growing use of LLMs to give quick explanations and summaries of long and complex medical scenarios can therefore be used to generate unique patient cases and practice questions and can also be applied to develop educational curricula and other educational materials specific to the learner [37,38]. Despite this promising landscape, the current evidence base remains predominantly unvalidated or derived from adult simulation environments that do not fully replicate the complexity of pediatric emergency settings; future research should therefore prioritize prospective, real-world studies evaluating AI-driven educational interventions in actual PED trainees, using measurable learning outcomes or clinical performance endpoints to determine whether these tools translate into meaningful improvements in emergency care clinical practice. Collectively, the literature highlights a shared responsibility among AI developers, academic institutions, students, pediatric educators, researchers, and community pediatricians to ensure that ML integration into pediatric emergency training could be rigorously validated, ethically governed, and equitably implemented—particularly given disparities in resource access across settings—so that all stakeholders contribute to and benefit from tools that ultimately improve pediatric emergency outcomes [34,36].

**Figure 1:**
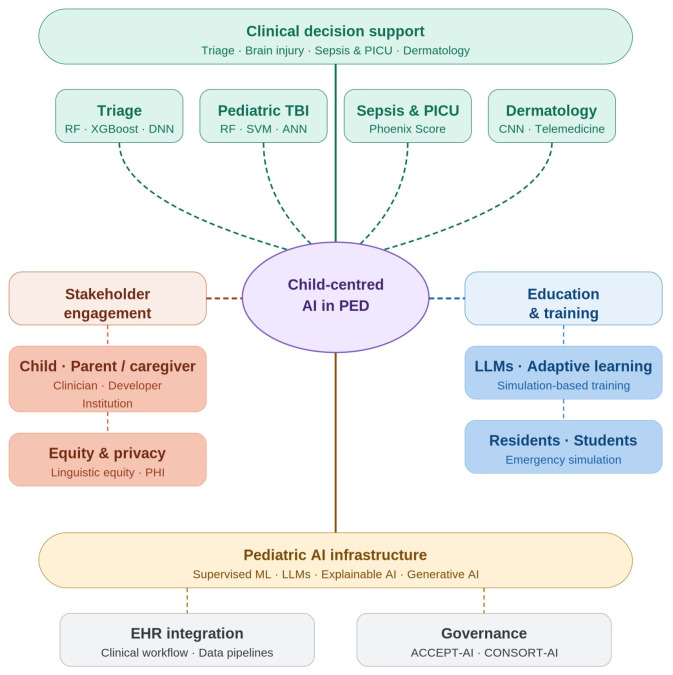
Conceptual framework of child-centered AI in the PED, illustrating three interconnected domains—clinical decision support, stakeholder engagement (including children, parents/caregivers, clinicians, developers, and institutions), and education & training—all grounded in a shared pediatric AI infrastructure.

To provide an integrated overview of the applications discussed in this section, Figure 1 presents a conceptual framework of child-centered AI in the PED, highlighting the interconnection between clinical decision support, stakeholder engagement, and education and training.

## 4. Discussion

### 4.1. Limitation of AI

#### 4.1.1. Data Quality and Data Standardization

The widespread adoption of EHRs has sparked a data revolution, making it possible to leverage a vast amount of data even within a chaotic setting such as a pediatric emergency room. The data within the EHRs reflects local daily clinical practice, incorporating a level of granularity resulting from different workflows, specializations and cultures. Despite the need for standardization in the use of EHRs, this level of detail best captures the relevant characteristics of the population being described (for example, in terms of age, gender, sex, ethnicity, geographic location, and medical conditions) and of the various stakeholders involved in the care of pediatric patients [39,40]. For each variable included in the dataset, it is essential to check three main categories: conformance, completeness, and plausibility. Conformance addresses whether the data is in the expected format; completeness, whether the data represents the observation; and plausibility, whether the data is reliable. In addition to these three criteria, it is undoubtedly necessary to consider the relevance of the data: is the data relevant to the desired observation [41]? Other key characteristics of ML models include generalizability, reproducibility, and implementability. Generalizability assesses how well the model performs in different settings. Reproducibility, on the other hand, assesses the model’s ability to produce similar results under similar conditions. Generalizability may be undermined when models rely on inputs with limited real-world availability, while reproducibility can be compromised by data noise and practically irreproducible preprocessing pipelines. A further concern pertains to the temporal availability of predictors: variables assumed present at prediction time—such as admission diagnosis codes in electronic health records—may in practice only be recorded after the clinical event, introducing temporal data leakage that degrades deployment performance. Implementability is the application into usual healthcare environment [40,42].

#### 4.1.2. AI Governance Guidelines

Within the broad advancement of AI in healthcare, it is essential to establish shared principles to promote safe, unbiased, and high-quality development. At every stage of AI development, it is paramount to uphold the core ethical principles of beneficence, non-maleficence, transparency, inclusiveness, and equity. Over the years, numerous guidelines for AI in healthcare and research have been developed and formalized, including CONSORT-AI, SPIRIT-AI, TRIPOD-AI, and PROBAST-AI. However, none of these frameworks is specifically tailored to the pediatric setting. The absence of pediatric-specific guidelines introduces the risk of age-related algorithmic bias, arising from the underrepresentation of pediatric populations in adult ML models and the inappropriate extrapolation of adult-derived datasets to pediatric contexts, or vice versa. ACCEPT-AI represents a proposed guidance framework specifically intended for application in pediatric healthcare. It advocates that all pediatric ML research should systematically address the following core domains: age, parental consent and subject assent, communication, equity, data protection, and identification. It stands as one of the first guidance frameworks specifically developed for AI/ML research in the pediatric age group, conceived as a complement to the aforementioned established reporting guidelines. However, its adoption remains limited, and it is not yet listed on the EQUATOR network, the international network dedicated to promoting transparent and high quality reporting of health research. This absence is an indicator of the early-stage maturity of pediatric AI governance frameworks and represents an urgent need for broader dissemination, formal recognition and rigorous validation of ACCEPT-AI as a standard reporting tool for pediatric ML research [40,43]. The need for governance frameworks specific to pediatric clinical practice and research is fundamental to enabling the safe, ethical, and effective implementation of ML in pediatric care—one that is responsive to the distinct developmental trajectories and vulnerabilities of this population. This requires fostering investment and institutional infrastructure, enabling the creation of high-quality shared datasets, engaging all relevant stakeholders—including caregivers, pediatricians, healthcare providers, and children themselves—ensuring equitable access, and facilitating pediatric-specific evaluation methodologies [43].

## 5. Conclusions and Future Directions

**Figure 2:**
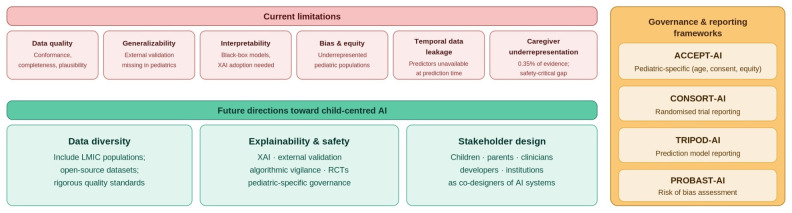
Overview of current limitations, governance frameworks, and future strategic directions for the implementation of artificial intelligence in the PED.

AI has the potential to determine a paradigm shift in pediatric emergency medicine, analogous to what is already occurring across other medical specialties. As demonstrated in the literature, numerous ML models have proven technically equivalent to or superior to traditional methods and have shown utility as clinical decision support tools; however, the majority of these models have been implemented from retrospective dataset in single-center studies, and none has yet proven suitable for real-world implementation in routine clinical practice. The future of AI in pediatric practice must be shaped in accordance with the rights and well-being of the child, not merely transposing from adult algorithms. To enable the broadest possible generalizability, it is essential to promote data diversity within the pediatric population, with greater inclusion of currently underrepresented groups such as those from low- and middle-income countries, and to engage researchers from diverse backgrounds and institutions, fostering open-source models and secure data sharing. It is imperative to ensure explainability, external validation, and safety monitoring through algorithmic vigilance methodologies, alongside real-time updates and continuous model refinement. Supporting research, the implementation of randomized controlled clinical trials, and the adoption of pediatric-specific governance frameworks are equally fundamental priorities. Furthermore, the involvement of all stakeholders—children, parents, clinicians, and developers—is mandatory and a non-negotiable foundation throughout the design and development of pediatric AI systems, enabling precision medicine even within emergency settings and effectively supporting clinical decision-making. Several limitations of the present work should be acknowledged. As a perspective article, this work does not employ formal inclusion criteria or structured evidence grading; the strength of evidence supporting each cited AI application is heterogeneous, and the discussion reflects the authors’ interpretive synthesis rather than a systematic appraisal. The literature reviewed is representative but not exhaustive, and potential publication bias toward studies reporting positive AI performance may have influenced the overall framing. Despite these limitations, as summarized in Figure 2, this perspective offers a synthesis of the current landscape of pediatric emergency medicine, and aims to serve as a foundation for more rigorous and pediatric-specific research in this evolving field.

## Data Availability

No new data were created or analyzed in this study. Data sharing is not applicable to this article.

## Abbreviations

The following abbreviations are used in this manuscript: Pediatric Emergency Departments (PEDs); Electronic Health Records (EHRs); Artificial Intelligence (AI); Machine Learning (ML); Pediatric Intensive Care Unit (PICU); Traumatic Brain Injury (TBI).
